# A Protein/Lipid Preload Attenuates Glucose-Induced Endothelial Dysfunction in Individuals with Abnormal Glucose Tolerance

**DOI:** 10.3390/nu12072053

**Published:** 2020-07-10

**Authors:** Domenico Tricò, Lorenzo Nesti, Silvia Frascerra, Simona Baldi, Alessandro Mengozzi, Andrea Natali

**Affiliations:** 1 Department of Surgical, Medical and Molecular Pathology and Critical Care Medicine, University of Pisa, Via Savi 10, 56126 Pisa, Italy; 2 Institute of Life Sciences, Sant’Anna School of Advanced Studies, Via Santa Cecilia 3, 56127 Pisa, Italy; 3 Department of Clinical and Experimental Medicine, University of Pisa, Via Roma 67, 56126 Pisa, Italy; lorenzonesti90@gmail.com (L.N.); lafrasce@gmail.com (S.F.); simona.baldi@dmi.unipi.it (S.B.); alessandro.mengozzi@mcs.unipi.it (A.M.); andrea.natali@med.unipi.it (A.N.)

**Keywords:** amino acids, arginine, endothelial function, GLP-1, impaired glucose tolerance, protein preload, type 2 diabetes, vascular reactivity

## Abstract

Postprandial hyperglycemia interferes with vascular reactivity and is a strong predictor of cardiovascular disease. Macronutrient preloads reduce postprandial hyperglycemia in subjects with impaired glucose tolerance (IGT) or type 2 diabetes (T2D), but the effect on endothelial function is unknown. Therefore, we examined whether a protein/lipid preload can attenuate postprandial endothelial dysfunction by lowering plasma glucose responses in subjects with IGT/T2D. Endothelial function was assessed by the reactive hyperemia index (RHI) at fasting, 60 min and 120 min during two 75 g oral glucose tolerance tests (OGTTs) preceded by either water or a macronutrient preload (i.e., egg and parmesan cheese) in 22 volunteers with IGT/T2D. Plasma glucose, insulin, glucagon-like peptide-1 (GLP-1), glucose-dependent insulinotropic polypeptide (GIP), glucagon, free fatty acids, and amino acids were measured through each test. RHI negatively correlated with fasting plasma glucose. During the control OGTT, RHI decreased by 9% and its deterioration was associated with the rise in plasma glucose. The macronutrient preload attenuated the decline in RHI and markedly reduced postprandial glycemia. The beneficial effect of the macronutrient preload on RHI was proportional to the improvement in glucose tolerance and was associated with the increase in plasma GLP-1 and arginine levels. In conclusion, a protein/lipid macronutrient preload attenuates glucose-induced endothelial dysfunction in individuals with IGT/T2D by lowering plasma glucose excursions and by increasing GLP-1 and arginine levels, which are known regulators of the nitric oxide vasodilator system.

## 1. Introduction

Despite the global improvement in diabetes care and the large availability of novel therapies, type 2 diabetes (T2D) and the prediabetic condition remain associated with increased cardiovascular risk [1]. This excess risk can be partly attributed to the adverse effects of hyperglycemia and glucose-induced oxidative stress on normal vascular biology [2,3,4,5]. Vascular endothelial cells secrete several mediators that participate in the regulation of vascular tone, platelet aggregation, coagulation, and fibrinolysis. The loss of one major physiologic property of the endothelium, which is the ability to induce vascular relaxation in response to transient ischemia, is commonly used to identify the condition of “endothelial dysfunction”, an early driver of atherosclerosis and cardiovascular disease [6].

Postprandial hyperglycemia occurs early in the progression of T2D and is a stronger predictor of cardiovascular disease than fasting hyperglycemia [7,8]. The acute increase in plasma glucose levels in response to carbohydrate ingestion rapidly and transiently suppresses endothelium-mediated vasodilation in a dose-dependent fashion [8,9,10]. Proposed mechanisms of endothelial dysfunction related to postprandial hyperglycemia and diabetes involve alterations in arginine [11], branched chain amino acid (BCAA) [12], and free fatty acid (FFA) metabolism [13], and changes in gut hormones such as the glucagon-like peptide-1 (GLP-1) [14]. In metabolically healthy individuals, the negative effect of postprandial hyperglycemia on endothelial function is counterbalanced by a parallel increase in insulin-induced nitric oxide (NO) production by endothelial cells. This compensatory mechanism, however, is reduced in insulin-resistant states [15]. A better understanding of the factors modulating vascular dilation in the absorptive phase and the identification of non-pharmacological strategies that positively impact on postprandial endothelial dysfunction are needed to improve the management of prediabetes and T2D.

We [16,17,18] and others [19,20,21,22,23] have recently demonstrated that a protein/lipid preload, ingested shortly before an oral glucose load, markedly reduces postprandial glucose responses by delaying oral glucose absorption and by increasing plasma amino acids, gut hormones, and glucose-stimulated insulin secretion [24,25]. Hence, we hypothesized that a similar nutritional approach could prevent the deterioration of endothelial function induced by glucose ingestion in individuals with prediabetes or T2D. To test this hypothesis, in the present study we assessed fasting and postprandial endothelial function after oral glucose loading preceded by water or a protein/lipid preload in subjects with abnormal glucose tolerance (AGT). We also measured plasma substrates and hormones to dissect the underlying physiological mechanisms.

## 2. Materials and Methods

### 2.1. Subjects

A total of 35 volunteers were recruited among students, fellows and patients attending the outpatient clinic of the Unit of Clinical Nutrition and Dietetics at the University of Pisa (Italy). The inclusion criteria were age 18–65 years, body mass index (BMI) 18–35 kg/m^2^, both men and women. Individuals with chronic or acute diseases (other than diet-controlled T2D and overweight/obesity), taking medications influencing endothelial function or glucose metabolism, and pregnant women were excluded. Individuals with T2D were recruited if recently diagnosed (≤5 years) and adequately controlled with diet alone (glycated hemoglobin 48–58 mmol/mol for at least six months). For the purpose of this study, former smokers were included if they had quit smoking for at least five years, while active smokers were excluded (*n* = 5). This resulted in a study population of 30 subjects.

Information regarding medical history, drug use, and smoking status was collected using standardized self-reported questionnaires. Brachial blood pressure was measured three times in subjects seated for at least 10 min, and the last two measurements were averaged for analysis. Subjects were classified as having normal glucose tolerance (NGT) or AGT, according to the current diagnostic criteria [26]. The latter group included individuals with either impaired glucose tolerance (IGT) or T2D.

The study was approved by the local Human Ethics Committee (Comitato Etico di Area Vasta Nord Ovest, or CEAVNO, clinical trial number: NCT02342834, approval code 13053_NATALI) and conducted in accordance with the principles expressed in the Declaration of Helsinki. All subjects provided written informed consent before enrollment.

### 2.2. Study Design

**Figure 1:**
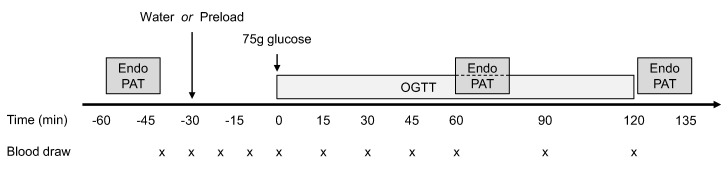
Outline of the study protocol. Endothelial function was assessed by the reactive hyperemia index (RHI) using an EndoPAT device at fasting, 60 min and 120 min during two 75 g oral glucose tolerance tests (OGTTs) preceded by water or a protein/lipid preload. Plasma glucose, insulin, glucagon-like peptide-1 (GLP-1), glucose-dependent insulinotropic polypeptide (GIP), glucagon, free fatty acids, and amino acids were measured.

An outline of the study protocol is depicted in Figure 1. In this open, cross-over, randomized controlled trial, we measured fasting and postprandial endothelial function in individuals with AGT during two oral glucose tolerance tests (OGTTs) preceded by either water or a non-carbohydrate macronutrient preload, in a random order. Endothelial function was also measured during a control OGTT preceded by water in people with NGT. Further details of the protocol and metabolic data from the original cohort (*n* = 35) have been previously reported [16,27].

### 2.3. Metabolic Tests

Metabolic procedures were performed after an overnight fast (12 h) on two days separated by 2–4 weeks. Participants were asked to maintain their habitual lifestyle and to refrain from alcohol, caffeine, and exercise for at least 24 h prior to each visit. On each study day, volunteers were admitted to our Clinical Research Unit at 8 am. The tests were performed in a quiet room with controlled temperature of 20–22 °C. A 20-gauge polyethylene cannula was inserted into a superficial vein of the upper limb for blood sampling. Thirty minutes before glucose ingestion (time −30 to −25 min), volunteers were randomized to consume either 500 mL water (control study) or a small macronutrient preload consisting of 50 g parmesan cheese, one boiled egg, and 300 mL water (preload study). The preload was rich in protein and fat and virtually free from carbohydrates (23 g protein, 17 g fat, 2 g carbohydrate, for a total of ~250 Kcal). A glucose drink consisting of 150 mL of 50% glucose solution (wt/vol) was consumed at time 0. Blood samples were collected during each test at time −40, −30, −20, −10, 0, 15, 30, 45, 60, 90, and 120 min to measure plasma glucose, insulin, GLP-1, GIP, glucagon, free fatty acids (FFA), and amino acids (AA).

### 2.4. Peripheral Arterial Tonometry (PAT)

Endothelial function was assessed at fasting (time −60), at 60 min and at 120 min during each OGTT using an EndoPAT device (EndoPAT 2000, Itamar Medical Ltd., Caesarea, Israel), according to standard procedures [28,29,30]. This device records changes in the digital pulse waveform elicited by a downstream hyperemic response to transient ischemia, which are largely dependent on proper endothelial function [29]. Room temperature was maintained as stable throughout the study. Peripheral arterial tonometry (PAT) probes were placed on both forefingers for continuous recording of the PAT signal. An inflatable blood pressure cuff was placed on the dominant upper arm. After a 5-min equilibration period, the cuff was inflated to suprasystolic pressures for 5 min. Then the cuff was deflated, while PAT recording continued for 5 min. The reactive hyperemia index (RHI) was calculated automatically by the EndoPAT software (software version 3.1.2, Itamar Medical Ltd., Caesarea, Israel) as the ratio between post- and pre-occlusion amplitudes of the PAT signal, normalized to the contralateral finger [28,29,30]. In addition, the EndoPAT technique allows the estimate of arterial stiffness by calculating the augmentation index (AI) from the analysis of the pulse-wave contour. AI is automatically calculated as the ratio of the difference between the late (P2) and early (P1) systolic peaks of the waveform relative to the early peak (P2–P1/P1), expressed as a percentage. To adjust for differences in heart rate, EndoPAT also records heart rate during the measurement and provides an AI normalized to heart rate of 75 bpm (AI@75).

### 2.5. Analytical Methods and Calculations

Plasma glucose was measured at the bedside by the glucose-oxidase technique (Beckman Glucose Analyzer II, Beckman Instruments, Fullerton, CA, USA). Insulin and C-peptide assays were performed by electrochemiluminescence on a COBAS e411 instrument (Roche, Indianapolis, IN, USA). Plasma GLP-1, GIP and glucagon were assayed using a multiplex immunoassay (Biorad Laboratories, Hercules, CA, USA). Plasma FFA were assayed by standard spectrophotometric methods on a Synchron Clinical System CX4 (Beckman Instruments). Plasma amino acids were measured in all AGT subjects with available blood samples (*n* = 10) using a reverse-phase, high-performance liquid chromatography system (HPLC), as previously described [27]. For the purposes of this study, arginine, branched chain amino acids (BCAA; i.e., leucine, isoleucine, and valine) and total amino acids data were analyzed. To estimate insulin sensitivity, we calculated the Homeostatic Model Assessment for Insulin Resistance (HOMA-IR) index, the Matsuda index, the Oral Glucose Insulin Sensitivity index (OGIS), and the hepatic insulin resistance index (HIRI) [31,32].

### 2.6. Statistical Analysis

Group differences were analyzed by Kruskal-Wallis test for continuous variables and Fisher exact test for categorical variables, followed by post-hoc pairwise comparisons as appropriate. In AGT, repeated measures were analyzed by Wilcoxon signed-rank test or by mixed models including the variable of interest, time, and the interaction between variable and time as fixed effects and subject as random effect. To account for potential sex-related differences in men and women, sex and interaction factors were also added to multivariable models.

Variables with a skewed distribution were log-transformed before mixed model analysis. Bivariate correlations were tested using Kendall’s correlation. Three subjects had missing data (RHI at 120 min during one study visit due to technical reasons) and therefore were excluded from correlation analyses. Areas under the curve (AUC) were calculated using the trapezoidal rule. Data are presented as mean ± SD or median [interquartile range], unless otherwise stated. RHI data during the OGTT are presented as percentage changes from baseline to adjust for the inter- and intra-individual variability of baseline values. Based on previous data [28], a sample size of 22 subjects was calculated to provide 80% power to detect a difference of at least 5% in RHI at the end of the OGTT between the preload and control study, deemed clinically significant (α = 0.05, two-sided). All tests were conducted at a two-sided α level of 0.05. Analyses were performed using JMP Pro software version 13.2.1 (SAS Institute, Cary, NC, USA).

## 3. Results

### 3.1. Fasting and Post-Glucose Endothelial Function

The clinical and metabolic characteristics of study participants stratified by glucose tolerance status are shown in Table 1.

**Figure 2:**
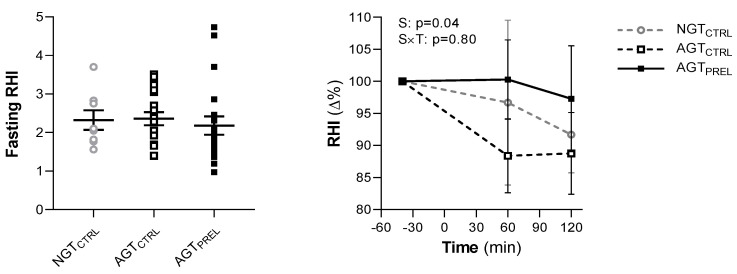
Fasting endothelial function assessed by the reactive hyperemia index (RHI) and its percentage changes (Δ%) during two 75 g oral glucose tolerance tests preceded by water (CTRL) or a protein/lipid preload (PREL) in subjects with normal glucose tolerance (NGT) or abnormal glucose tolerance (AGT). Data are mean ± SEM. Baseline differences between the three groups were tested by Kruskal–Wallis test followed by post-hoc pairwise comparisons. In AGT, repeated measures were analyzed by mixed models including study (S), time (T), and an interaction term (S × T) as fixed effects and subject as random effect. *p* value for time effect is <0.05.

At fasting, the average RHI was 2.35 ± 0.70. Five individuals had an RHI lower than 1.67, indicative of endothelial dysfunction [28], including four people with AGT and one with NGT (*p* = 0.56 for group differences). RHI was similar between men and women (2.36 ± 0.70 vs. 2.34 ± 0.74, respectively, *p* = 0.94) and across groups of glucose tolerance (Figure 2). RHI was not associated with age, BMI, or systolic and diastolic blood pressure. Among metabolic variables, RHI negatively correlated with fasting plasma glucose (*r* = −0.29, *p* = 0.04) and HOMA-IR (*r* = −0.29, *p* = 0.04), and positively correlated with Matsuda index (*r* = 0.39, *p* = 0.048). RHI was not significantly associated with fasting plasma insulin (*r* = −0.25, *p* = 0.07), nor with OGIS, HIRI, GLP-1, GIP, glucagon, FFA, or total AA.

**Figure 3:**
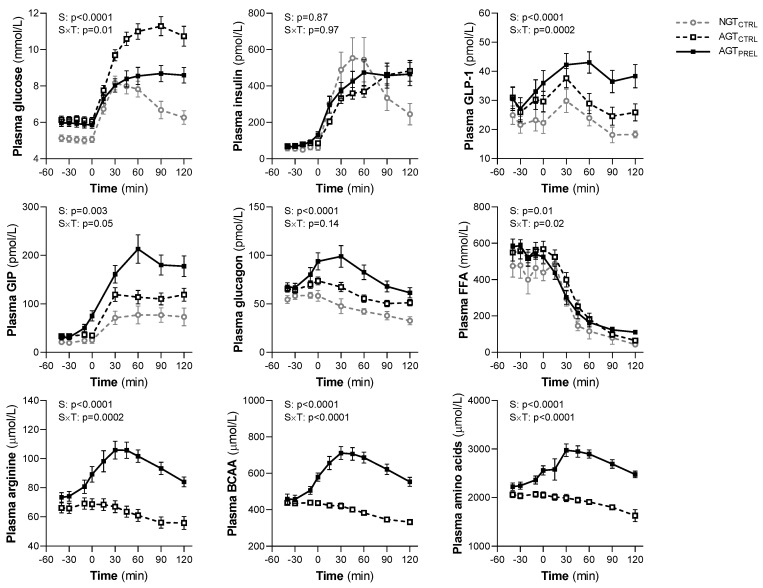
Plasma concentrations of glucose, insulin, glucagon-like peptide-1 (GLP-1), glucose-dependent insulinotropic polypeptide (GIP), glucagon, free fatty acids (FFA), arginine, branched-chain amino acids (BCAA), and total amino acids during two 75 g oral glucose tolerance tests preceded by water (CTRL) or a protein/lipid preload (PREL) in subjects with normal glucose tolerance (NGT) or abnormal glucose tolerance (AGT). Data are mean ± SEM. In AGT, repeated measures were analyzed by mixed models including study (S), time, and an interaction term (S × T) as fixed effects and subject as random effect. P values are not shown for time effects (<0.05 for all variables).

After glucose ingestion, we observed a significant decrease in RHI, with a median 9% reduction from baseline values at 120 min in all participants (*p* = 0.02; *p* = 0.74 for group difference) (Figure 2). The time course of RHI was similar across glucose tolerance groups (*p* = 0.72; group × time interaction: *p* = 0.60) and by sex (*p* = 0.54; sex × time interaction: *p* = 0.23). As expected, all measured hormones and metabolites were affected by the OGTT (Figure 3). In repeated measure analyses, RHI values during the OGTT were associated with plasma glucose (β = −0.03, *p* = 0.015) and insulin levels (β = −0.10, *p* = 0.01). The RHI was not related to plasma GLP-1, GIP, glucagon, FFA, or AA levels throughout the OGTT.

### 3.2. Effect of Nutrients on Post-Glucose Endothelial Dysfunction

On a separate day, in random order, endothelial function was assessed during an OGTT preceded by the protein/lipid macronutrient preload in individuals with AGT. Fasting RHI measurements were similar between the two study days (*p* = 0.45; coefficient of variation 26.4%) (Figure 2).

Compared with the control OGTT, the postprandial reduction of RHI was significantly attenuated by nutrient ingestion (Figure 2), without sex differences. In parallel, plasma glucose excursion was markedly reduced by nutrients (AUC 982 ± 186 vs. 1213 ± 203 mmol × min/L, *p* < 0.0001), while plasma insulin responses were similar between the two studies (AUC 38.0 [25.9–60.2] vs. 38.9 [31.5–50.7] nmol × min/L, *p* = 0.40) (Figure 3). The time courses of all other measured hormones and metabolites showed significant differences compared with the control OGTT (Figure 3).

**Figure 4:**
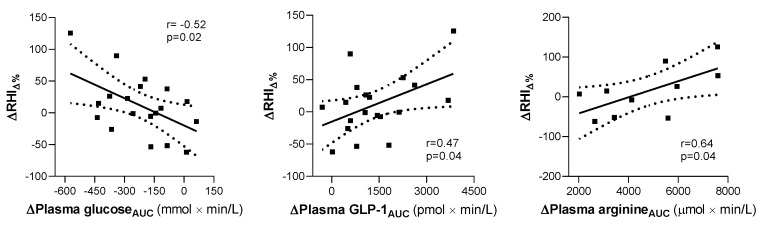
Correlations between differences in RHI percentage changes at the end of the preload vs. control study (ΔRHI_Δ%_) and changes in the areas under the curve (AUC) of plasma glucose (Δglucose_AUC_), glucagon-like peptide-1 (ΔGLP-1_AUC_), and arginine (Δarginine_AUC_) in subjects with abnormal glucose tolerance.

The attenuation of postprandial endothelial dysfunction induced by the macronutrient preload was proportional to the effect on plasma glucose excursions (*r* = −0.52, *p* = 0.02) (Figure 4). In fact, RHI was reduced less in individuals whose glucose tolerance improved more after preload consumption. Furthermore, smaller or positive changes in RHI were associated with increased plasma GLP-1 (*r* = 0.47, *p* = 0.04) and arginine (*r* = 0.64, *p* = 0.04) levels during the preload study (Figure 4). Changes in other hormones and metabolites did not correlate with changes in RHI between the control and preload OGTT.

### 3.3. Fasting and Post-Glucose Arterial Stiffness

**Figure 5:**
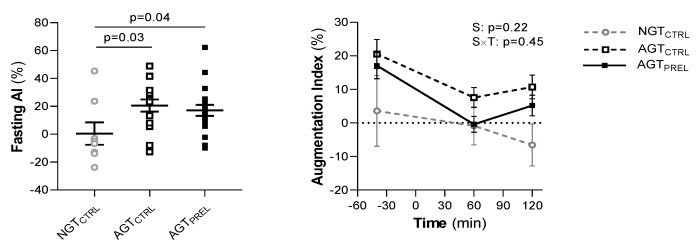
Fasting arterial stiffness assessed by the augmentation index (AI) and its change during two 75 g oral glucose tolerance tests preceded by water (CTRL) or a protein/lipid preload (PREL) in subjects with normal glucose tolerance (NGT) or abnormal glucose tolerance (AGT). Data are mean ± SEM. Baseline differences between the three groups were tested by Kruskal–Wallis test followed by post-hoc pairwise comparisons. In AGT, repeated measures were analyzed by mixed models including study (S), time (T), and an interaction term (S × T) as fixed effects and subject as random effect. *p* value for time effect is <0.05.

At fasting, the AI was significantly lower in NGT than AGT (*p* = 0.03, Figure 5) and positively correlated with age (r = 0.51, *p* = 0.01). The AI decreased in all subjects during the control OGTT (*p* < 0.0001 for 120 min vs. baseline values) (Figure 5). Its reduction was associated with plasma glucose (β = −3.4, *p* = 0.04) and tended to be greater in AGT than NGT (*p* = 0.07; group × time interaction: *p* = 0.18). Fasting and post-glucose AI were similar in men and women and not associated with other measured clinical or metabolic variables. In AGT, the AI was similar between the two study days at fasting (*p* > 0.99; coefficient of variation 37.1%) and its post-glucose decline was not attenuated by the macronutrient preload (Figure 5).

Glucose-induced changes in heart rate and AI@75 are shown in Figure S1 (Supplementary Materials). During the OGTT, the heart rate increased and the AI@75 decreased to a similar extent in NGT and IGT. Heart rate and AI@75 responses to glucose were not affected by the macronutrient preload (Figure S1).

## 4. Discussion

In this cross-over, randomized clinical trial, we demonstrated that a protein/lipid preload is able to prevent the acute endothelial dysfunction produced by glucose ingestion in individuals with IGT and early T2D. This effect of the macronutrient preload is likely to be explained by the more favorable glucose-to-insulin ratio and by the increase in plasma GLP-1 and arginine levels, both of which can upregulate the NO vasodilator system. To our knowledge, this is the first study that examined the effect of a high-protein/high-lipid macronutrient preload on postprandial endothelial dysfunction, revealing its potential mediators in a well-characterized cohort of individuals with IGT and well-controlled T2D. Our results have been obtained in an experimental setting that is easily transposable into real-life. Indeed, glucose loads are commonly provided by sugar-sweetened beverages (e.g., cola, orange juice), which contain on average 30–40 g carbohydrate (with no protein and fat) and do exert detrimental effects on endothelial function [33]. Further, there is compelling evidence that tailoring the sequence of nutrient consumption in the context of a mixed meal, so as to eat protein- and fat-rich food before carbohydrate, can improve postprandial glucose control to a similar extent compared with a preload approach [21,23,25]. Therefore, one might speculate that such a simple dietary strategy, if applied into a real-life setting, could have protective effects also on endothelial function in patients with prediabetes or manifest T2D.

In agreement with previous studies using different techniques to explore endothelial function [2,3,8,9,10], we found that RHI is acutely impaired by a physiologic plasma glucose rise in both NGT and AGT subjects. Although there is no conclusive demonstration that RHI is entirely dependent on the release of NO from endothelial cells, an elegant study has shown that at least 50% of this response depends on NO bioavailability [34]. RHI, in addition, has been validated against other procedures (i.e., flow mediated dilation, or FMD) for which a role of NO has been demonstrated [35] and correlates with erectile dysfunction [36], a clinical condition that is closely related to NO pathway impairment. With these limitations, it is plausible to hypothesize that an interference of the postprandial glucose increase with NO bioavailability explains the results of this study.

Crucial for endothelium-dependent vasodilation, NO is produced from L-arginine via the constitutively active, calcium-calmodulin dependent enzyme endothelial nitric oxide synthase (eNOS) [37]. Both chronic and acute hyperglycemia are known to induce uncoupling of eNOS from NO production in favor of superoxide. Moreover, NO availability is further reduced by superoxide combination with NO to form the highly reactive oxygen species peroxinitrite [38], which in turn further impairs eNOS activity starting a vicious cycle [39]. Also NADPH oxidases-membrane-bound enzyme complexes are considered an additional source of reactive oxygen species in endothelial cells, particularly under conditions of hyperglycemia [40], and asymmetric dimethylarginine, a naturally occurring product of amino acid metabolism that can bind eNOS [41], further interferes with NO availability.

We previously demonstrated that a high-protein nutrient preload is able to reduce the plasma glucose response to an OGTT largely by delaying gastric emptying [16,25]. In this view, it is of interest that other dietary manipulations able to delay gastric emptying have been reported to reduce the postprandial decline in conductance arteries’ endothelial function as measured by FMD [42]. Other glucose-lowering mechanisms activated by nutrient preloads include increased insulin secretion relative to plasma glucose and decreased insulin clearance, whose effects are partly counterbalanced by increased glucagon release and less suppressed endogenous glucose production [16,25].

Since carbohydrate ingestion increases simultaneously, and proportionally, both plasma glucose and insulin levels, it is challenging to dissect the role of hyperglycemia vs. hyperinsulinemia on endothelial function [10]. In healthy individuals, insulin activates eNOS-mediated production of NO and therefore exerts vasodilatory effects both on large conduit arteries [43] and resistance arterioles [44]. This hemodynamic effect is attenuated in conditions of insulin resistance or chronic hyperinsulinemia [15,45]. Our intervention markedly reduced glucose levels effecting neither insulin levels nor insulin sensitivity. We can therefore explain the beneficial effect of the preload on postprandial RHI with the reduction in plasma glucose levels and the amelioration of the post-OGTT glucose-to-insulin ratio; a mechanism we have previously demonstrated in non-physiologic experimental conditions [46].

In our study, we also observed a positive correlation of RHI with nutrient-induced plasma GLP-1 rise. This gut hormone is released after a meal and has extra-glycemic positive effects on the cardiovascular system [14]. The infusion of exogenous GLP-1 has been shown to ameliorate endothelial function in T2D, possibly through an improvement in the endothelial antioxidant properties and a decrease of oxidative stress [14,47]. These effects are both indirect (i.e., insulin-mediated) and direct on the endothelium, which expresses specific receptors for GLP-1. In support of our findings, Tanaka et al. [48] demonstrated that RHI is unchanged 2 h after a mixed meal in 17 T2D patients treated with a single injection of exenatide, a short-acting GLP-1 receptor agonist, while it decreases by 15% without exenatide.

The improvement in endothelial function induced by the macronutrient preload might also be due to the greater availability of substrates entering the circulation following digestion and absorption of nutrients, such as amino and fatty acids. Among the measured substrates, only the increase in plasma arginine after the high-protein preload was correlated with the improvement in post-glucose endothelial function. Arginine plays a central role in the biosynthesis of NO, being its direct precursor, and an increased arginine bioavailability may facilitate NO production particularly in the stimulated condition of post-ischemic vasodilation. In fact, a reduction in arginine availability through increased arginase activity impairs NO production in T2D [11,49], while arginase inhibition ameliorates endothelial function [50]. Furthermore, the lack of the glucose-induced decline of plasma arginine when fructose—rather than glucose—is ingested [10] or when a small amount of protein is co-ingested with glucose [51] has been hypothesized to be responsible for the lesser impact of the oral load on endothelial function. Finally, the increase in plasma arginine might also counterbalance the rise in asymmetric dimethylarginine (ADMA) observed after glucose ingestion in subjects with AGT [52]. Dietary fats are known to exert a negative impact on postprandial endothelial function [53]. In our study, however, plasma FFA were similarly suppressed during the OGTTs preceded by water or nutrients and did not correlate with changes in RHI.

Measures of endothelial function are to some extent dependent on arterial stiffness and blood pressure, particularly when measuring acute responses [54,55]. Indeed, our estimates of stiffness (AI and AI@75) showed a decline after glucose consumption that was consistent with previous observations using mixed meals [56] and was not affected by the macronutrient preload. Although we did not measure blood pressure during the OGTTs, it has been observed by Pham et al. [57] that blood pressure responses to oral glucose are not different when glucose is preceded by a high-protein load. Most importantly, the RHI is automatically corrected for changes in systemic hemodynamics at each time point, being calculated as the post-to-pre occlusion PAT signal ratio in the occluded arm, relative to the same ratio in the control arm [28,29,30]. For these reasons, we consider changes in arterial stiffness and blood pressure unlikely to explain the observed changes in RHI.

This study has some limitations, such as the small sample size and the lack of repeated blood pressure measurements. Given that RHI is similarly reduced during the OGTT across glucose tolerance groups, nutrient preloads may also exert beneficial effects in NGT, which have not been verified. NGT had lower age and blood pressure than AGT. Both factors could influence endothelial function, and this should be considered when interpreting group differences. Also, our study was not powered to assess differences between the two groups of individuals with prediabetes and early diagnosed, diet-controlled diabetes. These groups were substantially homogeneous, representing two consecutive phases in the continuum of diabetes progression, and therefore combined data were analyzed and presented. The homogeneity of our population and the small sample size might also explain the lack of correlation between RHI and known factors associated with endothelial dysfunction (such as age, BMI and blood pressure), which were all demonstrated in large, population-based cohorts [30]. Participants were not characterized in terms of plasma lipid profile and waist circumference, which may also influence endothelial function. Our study design did not include a protein-only or a mixed-meal control group; thus, we cannot fully dissect the impact of protein alone or of the preload strategy on endothelial function. Preliminary evidence supports the sustained metabolic benefits of preload strategies and their neutral impact on body weight (likely as a consequence of reduced appetite after protein consumption) [17,20]. Due to the lack of longitudinal data, however, we could not examine the long-term efficacy of the preload strategy on endothelial function and its effects on body weight. Although the inclusion of both men and women strengthens the external validity of the present study, differences related to the sex and to different phases of menstrual cycle in pre-menopausal women may introduce variability. Moreover, despite the fact that AI is widely used in clinics and research as an indirect measure of arterial stiffness, it measures pulse wave reflections rather than vascular stiffness and can be influenced by several factors [58,59,60]. Finally, although the dependence of postprandial endothelial dysfunction on hyperglycemia-induced NO impairment relies on a strong biological rationale and is indirectly supported by our findings, NO availability and its metabolic signaling was not directly investigated in this study.

## 5. Conclusions

In conclusion, a protein/lipid macronutrient preload ameliorates acute glucose-induced endothelial dysfunction in individuals with early AGT. This effect is possibly mediated by an improved glucose-to-insulin ratio, as well as higher GLP-1 and arginine bioavailability. Our results support the efficacy of this dietary strategy to improve glucose control and prevent the deterioration of the endothelial vasodilator function induced by the hyperglycemic environment.

## Figures and Tables

**Table 1:** Clinical and metabolic characteristics of study participants.

	AGT	NGT	*p*
*N*	22	8	-
Age (years)	50.0 ± 14.2	31.8 ± 11.9	0.004
Sex (men/women; *n* (%))	14/8 (63.6/36.4)	4/4 (50.0/50.0)	0.68
Body Mass Index (kg/m^2^)	27.4 ± 5.5	26.6 ± 5.1	0.64
Systolic Blood Pressure (mmHg)	121 ± 9	109 ± 10	0.006
Diastolic Blood Pressure (mmHg)	78 ± 8	69 ± 8	0.01
Heart Rate (bpm)	63 ± 8	59 ± 10	0.45
Non-smokers/Ex-smokers (*n* (%))	17/5 (77.3/22.7)	7/1 (87.5/12.5)	0.99
Fasting Plasma Glucose (mmol/L)	6.0 ± 1.0	5.1 ± 0.5	0.02
2-h Plasma Glucose (mmol/L)	10.7 ± 2.6	6.3 ± 1.1	<0.0001
Plasma Glucose AUC (mmol × min/L)	1,213 ± 203	852 ± 105	0.0002
Glucose tolerance (IGT/T2D; *n* (%))	13/9 [59/41]	-	-
HbA1_c_ (%)	6.1 ± 0.6	5.3 ± 0.2	0.002
Fasting Plasma Insulin (pmol/L)	79 [42–120]	52 [27–75]	0.09
2-h Plasma Insulin (pmol/L)	398 [299–703]	192 [141–425]	0.02
Plasma Insulin AUC (nmol × min/L)	38.9 [31.5–50.7]	37.9 [30.6–59.8]	0.96
HOMA-IR (unit)	3.0 [1.4–4.7]	1.7 [0.8–2.7]	0.049
Matsuda Index (unit)	5.3 [3.4–9.6]	9.5 [5.1–14.1]	0.07
OGIS Index (unit)	357 [313–406]	416 [386–455]	0.003
HIRI (unit)	3.5 [2.6–4.4]	3.1 [2.4–6.5]	0.99

Data are mean  ±  SD or median [interquartile range] for normally or non-normally distributed variables, respectively. Differences were tested using Mann-Whitney test. Abbreviations: AGT, Abnormal Glucose Tolerance; AUC, Area Under the Curve; HbA1_c_, Glycated Hemoglobin; HIRI, Hepatic Insulin Resistance Index; HOMA-IR, Homeostatic Model Assessment for Insulin Resistance; IGT, Impaired Glucose Tolerance; NGT, Normal Glucose Tolerance; OGIS, Oral Glucose Insulin Sensitivity index; T2D, type 2 diabetes.

## Supplementary Materials

The following are available online at https://www.mdpi.com/2072-6643/12/7/2053/s1, Figure S1: Heart rate and augmentation index normalized to heart rate of 75 bpm (AI@75).

## Abbreviations

AA	Amino acid
AGT	Abnormal glucose tolerance
AI	Augmentation index
AI@75	AI normalized to heart rate of 75 bpm
AUC	Area under the curve
BCAA	Branched-chain amino acid
eNOS	Endothelial nitric oxide synthase
FFA	Free fatty acid
FMD	Flow mediated dilation
GIP	Glucose-dependent insulinotropic polypeptide
GLP-1	Glucagon-like peptide-1
HbA1_c_	Glycated hemoglobin
HIRI	Hepatic insulin resistance index
HOMA-IR	Homeostatic model assessment for insulin resistance
IGT	Impaired glucose tolerance
NGT	Normal glucose tolerance
NO	Nitric oxide
OGIS	Oral glucose insulin sensitivity
OGTT	Oral glucose tolerance test
RHI	Reactive hyperemia index
T2D	Type 2 diabetes
