# Supplementary material for: A Protein/Lipid Preload Attenuates Glucose-Induced Endothelial Dysfunction in Individuals with Abnormal Glucose Tolerance
Source: Nutrients. 2020 Jul 10;12(7):2053. doi: 10.3390/nu12072053 (PMC7400934; doi:10.3390/nu12072053)
Supplement: Supplementary file 1 [file nutrients-12-02053-s001.pdf]

Supplementary Materials

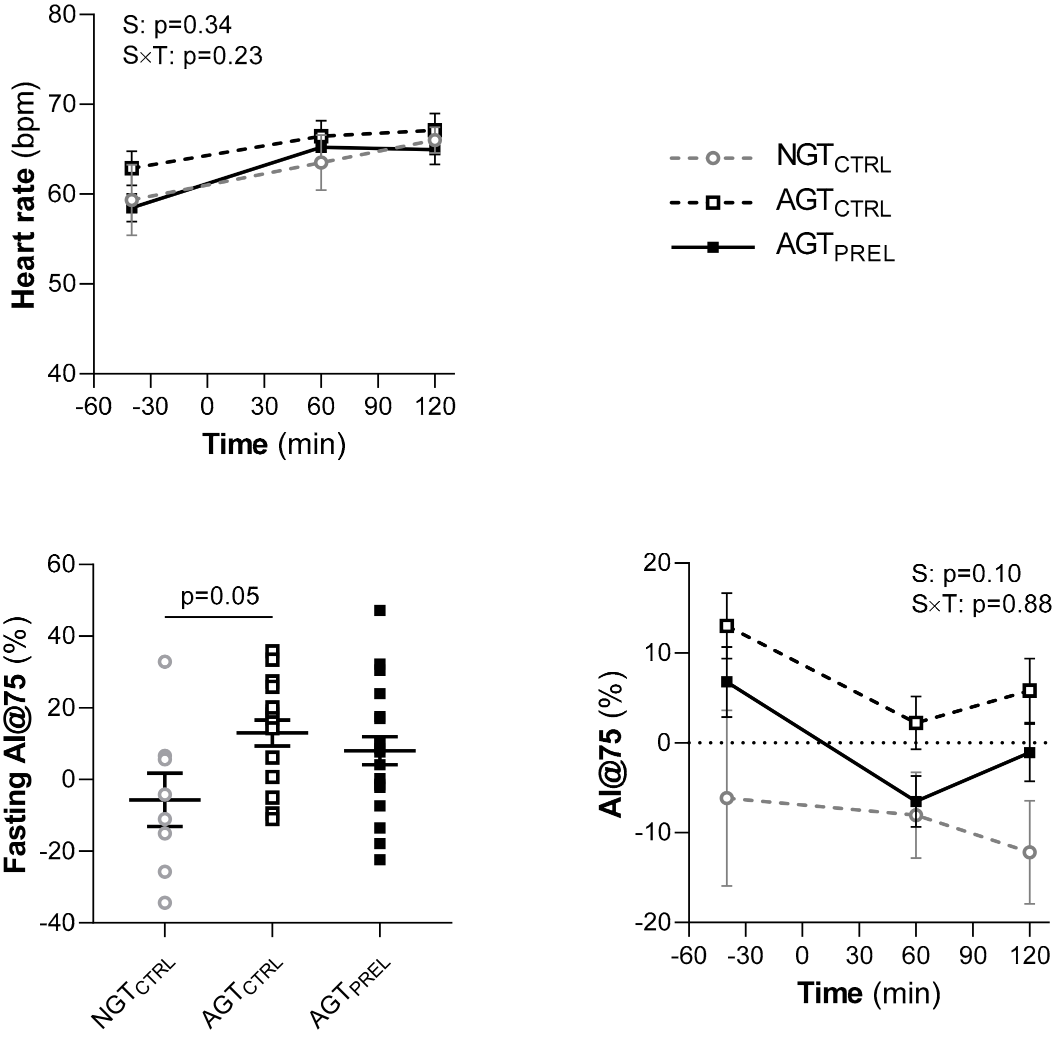

Figure S1. Heart rate, fasting arterial stiffness assessed by the augmentation index normalized to heart rate of 75 bpm (AI@75) and changes in AI@75 during two 75 g oral glucose tolerance tests preceded by water (CTRL) or a high-protein/non-carbohydrate preload (PREL) in subjects with normal glucose tolerance (NGT) or abnormal glucose tolerance (AGT). Data are mean ± SEM. Baseline group differences were tested by Kruskal–Wallis test followed by post-hoc pairwise comparisons. Repeated measures in AGT were analyzed by mixed models including study (S), time (T), and an interaction term (S×T) as fixed effects and subject as random effect. P values for time effects are not shown (<0.05 for all variables).
